# Biological System Responses of Dairy Cows to Aflatoxin B1 Exposure Revealed with Metabolomic Changes in Multiple Biofluids

**DOI:** 10.3390/toxins11020077

**Published:** 2019-02-01

**Authors:** Qian Wang, Yangdong Zhang, Nan Zheng, Liya Guo, Xiaoming Song, Shengguo Zhao, Jiaqi Wang

**Affiliations:** 1 State Key Laboratory of Animal Nutrition, Institute of Animal Science, Chinese Academy of Agricultural Sciences, Beijing 100193, China; sousawang@163.com (Q.W.); zhangyangdong@caas.cn (Y.Z.); zhengnan@caas.cn (N.Z.); gly2233@126.com (L.G.); songxiaoming311@163.com (X.S.); zhaoshengguo@caas.cn (S.Z.); 2 Key Laboratory of Quality & Safety Control for Milk and Dairy Products of Ministry of Agriculture and Rural Affairs, Institute of Animal Sciences, Chinese Academy of Agricultural Sciences, Beijing 100193, China; 3 Laboratory of Quality and Safety Risk Assessment for Dairy Products of Ministry of Agriculture and Rural Affairs, Institute of Animal Sciences, Chinese Academy of Agricultural Sciences, Beijing 100193, China

**Keywords:** aflatoxin B1, biofluid, metabolomics, dairy cow

## Abstract

Research on mycotoxins now requires a systematic study of post-exposure organisms. In this study, the effects of aflatoxin B1 (AFB1) on biofluids biomarkers were examined with metabolomics and biochemical tests. The results showed that milk concentration of aflatoxin M1 changed with the addition or removal of AFB1. AFB1 significantly affected serum concentrations of superoxide dismutase (SOD) and malon dialdehyde (MDA), SOD/MDA, and the total antioxidant capacity. Significant differences of volatile fatty acids and NH_3_-N were detected in the rumen fluid. Eighteen rumen fluid metabolites, 11 plasma metabolites, and 9 milk metabolites were significantly affected by the AFB1. These metabolites are mainly involved in the pathway of amino acids metabolism. Our results suggest that not only is the study of macro-indicators (milk composition and production) important, but that more attention should be paid to micro-indicators (biomarkers) when assessing the risks posed by mycotoxins to dairy cows.

## 1. Introduction

Mycotoxins contamination is a serious problem in farming and animal husbandry. In the 1960s, the occurrence of turkey X disease in the UK alerted farmers to the great danger posed by mycotoxins (aflatoxins). Subsequently, similar discovery led to growing public awareness of mycotoxins as food and feed contaminants that can cause illness and even death in both humans and animals [1,2,3]. With research progress in this field, it is now recognized that the mycotoxic contamination of feed is almost unavoidable [4]. So far, several hundred mycotoxins have been identified, and more than 25% of the world annual grain production is contaminated with mycotoxin [3,5].

Mycotoxins can affect animal health and their products to varying degrees. At the first level, the main manifestations of mycotoxins exposure in animals are reductions in feed intake and weight gain (Fink-Gremmels, 2008). The detoxification metabolism of the liver plays an important role in an animal’s resistance to mycotoxins, and this detoxification metabolism can be reflected in blood and/or urine parameters. Several specific biochemical parameters can be used to measure these metabolic processes [2,6,7]. At the second level, mycotoxins affect the quantity of animal products. Interestingly, several studies have shown that the effects of mycotoxins on milk yield are inconsistent [7,8]. The third level of influence is the safety and quality of the products from exposed animals [2,9,10]. Many studies have discussed the effects of mycotoxins on animals in terms of biochemistry and/or animal production. However, when only a small number of parameters are investigated; the findings will inevitably be biased. To ensure a comprehensive understanding of the harmful effects of mycotoxins, a multiple-level research strategy is used here.

Aflatoxins are mainly produced by the genus *Aspergillus*, and are commonly found in food and feed in humid and warm environments [1,3,11]. AflatoxinB1 (AFB1) was metabolized to aflatoxin M1 (AFM1) in the animal metabolic system. AFM1 appears in milk and also increases disease susceptibility [9,10,11,12]. These two types of aflatoxin are probably the well-known mycotoxins.

Studies have shown that milk composition, body mass gain, immunity, and reproductive performance are affected in dairy ruminants by feeds contaminated with aflatoxins [8,9,10]. However, an experiment in which lactating cows were fed AFB1-contaminated diets showed no significant reduction in milk production [13]. Dairy cows are considered more insensitive to mycotoxins than monogastric livestock because mycotoxins are readily degraded by rumen microbes [11,14]. However, some other studies have demonstrated that metabolic changes in rumen functions are caused by mycotoxins, including in volatile fatty acids (VFAs) and NH3-N [2,8]. The antioxidant capacities of biofluids can reflect the natural reactions of the body to various stressors. Diets contaminated with aflatoxins reduce the superoxide dismutase (SOD) enzyme activity in the blood and increase blood malon dialdehyde (MDA) levels in broiler chicks, whereas the addition of montmorillonite increases the blood SOD activity and reduces the levels of MDA [15]. Dairy goats administered multiple mycotoxins displayed lower serum SOD and total antioxidation competence (T-AOC), and higher MDA concentrations [7]. These data suggest that mycotoxins greatly affect the whole antioxidant capacity of animal [2]. However, current researches on aflatoxins in dairy ruminants has been basic and biochemically based, focusing on only one or several parameters [8,11,14]. The analysis of only a few biochemical parameters provides limited information and allows only simple metabolic inferences to be drawn. These limitations are most obvious in ruminant research. Therefore, a study of the overall metabolic mechanisms involved in the response of multiple-stomach herbivores, such as dairy cows, to mycotoxin exposure is urgently required [6,16].

Metabolomics is a systematic approach in the identification and quantification of the metabolites that directly reflect the organism’s systematic responses to endogenous or exogenous changes [17]. Nuclear magnetic resonance (NMR) spectroscopy has shown that the ratio of glycerophosphocholine to choline phosphate in cow milk in the first month of lactation can be used to determine the prognosis of ketosis [18]. Liquid chromatography–mass spectrometry (LC–MS) and NMR analyses identified 53 diagnostic biomarkers of heat stress in cow milk [19]. These studies suggest that the concentrations of metabolites present in milk can reflect the dairy cow’s performance under various circumstances. In this way, not only can the quality of dairy products be evaluated by measuring the levels of various metabolites in secreted biofluids, but the physiological or pathological conditions of the dairy cows can also be determined [6,16,20]. These methods have also been used in mycotoxin research.

In fact, AFB1 participates in and affects many physiological and biochemical processes [11,21]. Aflatoxin B1 exposure could changes in lipid oxidation, carbohydrate and amino acid metabolism in dairy goats [16]. Study has shown that these processes may require the participation of the cytochrome p450 system in liver [22]. The glutathione S-transferase system is an important metabolic pathway induced by detoxification of AFB1 in animal organisms [23]. AFB1 exposure can alter various metabolic pathways, including glycogenolysisand glycolysis in the process of carbohydrate metabolism [21], processes affecting phospholipid metabolism after aflatoxin B1 bind to DNA [24], and amino acid transport [23]. NMR-based metabolomics analysis is fast and reproducible, so it is widely used in the field of the toxicological investigation of mixed mycotoxins [25], single mycotoxins (ocharatoxin A) [26], and deoxynivalenol [27]. Based on the NMR and pattern recognition, researchers found that alpha-naphthylisothiocyanate changed energy metabolism characterized by increased plasma ketone bodies, and induced changes of various metabolic pathways, including hyperlipidaemia and hyperglycaemia. [28]. These studies have shown that blood, milk, urine, and tissue samples (liver, kidney, etc.) are important objects of research in the toxicological sciences. However, samples from a single source can only explain the unilateral metabolic status of the source. Therefore, samples must be acquired from different sources, in order to explain multiple simultaneous whole-body metabolic effects. Exposure to AFB1 causes changes in the metabolic processes in different organs and tissues [6]. The integration of data from different biofluids or tissue samples allows the systematic analysis of the toxic reactions within a global system [6,28].

AFB1 is commonly found in ruminant feeds and can lead to multiple toxicological effects throughout the body [1,2,3]. The study of AFB1 requires multiple samples and multiple methods to be considered simultaneously. Because AFB1 poses risks to animal health, product safety, and potentially human health, the maximum concentration allowed in feeds is limited. In China, the national hygiene standard for feeds limits AFB1 in feed to 20 μg/kg (also ppb) [29]. To understand the responses of biological systems to different levels of AFB1 exposure, we analyzed the metabolomic changes in multiple biological matrices (rumen fluid, blood, and milk) of dairy cows using 1H NMR spectroscopy and basic biochemical tests.

## 2. Results

### 2.1. Feed Intake, Milk Yield, and Milk Composition

**Figure 1:**
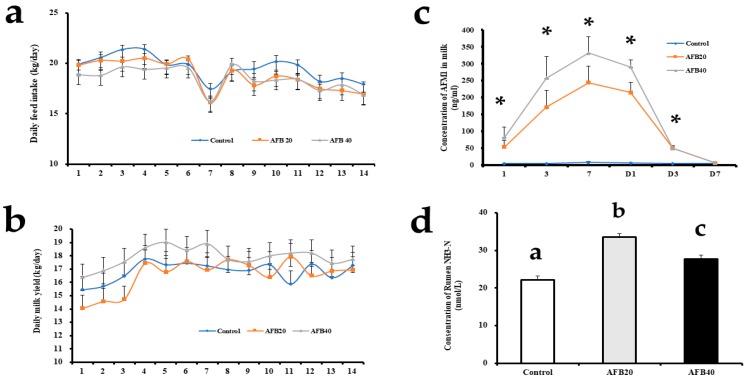
(**a**) Changes in the daily feed intake by dairy cows during addition or clearance of different doses of aflatoxin B1 (AFB1). (**b**) Changes in daily milk yield from dairy cows during addition or clearance of different doses of AFB1. (**c**) Changes in the aflatoxin M1 (AFM1) concentration in of milk during addition or clearance of different doses of AFB1.Asterisks “*” represent significant differences between groups. The x-axis indicates the time course of the experiment. (**d**) Effects of ingestion of AFB1-supplemented diet on concentration of rumen NH3-N. Control group (AFB1 null); AFB20 group (20 μg/kg in the total mixed ration); and AFB40 group (40 μg/kg in the total mixed ration). ^abc^ Means with different superscript letters are significantly different (*p* < 0.05), as determined with Tukey’s test.

There were no differences in the daily feed intake or milk yield among the control and treatment groups during the whole experiment (Figure 1a,b). The routine milk composition indices also showed no changes with treatment (*p* > 0.05) (Table 1).

### 2.2. Aflatoxin M1 Concentrations in Milk

The AFM1 concentrations in the milk of cows fed the 20 μg/kg or 40 μg/kg AFB1-contaminated diet were significantly higher than those in the control milk on day 1, 3, and 7 during the period of AFB1 addition (*p* < 0.05). After the cows stopped consuming the contaminated diets, the AFM1 concentrations still differed significantly on day 1 and 3 during the clearance period. However, there were no differences in the AFM1 concentrations on day 7 of the clearance period (Figure 1c).

### 2.3. Serum Biochemical Parameters

There were no significant differences in the serum parameters reflecting the liver and kidney functions or the immune functions (Table 2). However, there were significant differences between the control and two treatment groups in serum SOD activity, MDA, SOD/MDA, and T-AOC (all *p* < 0.05), but not in serum GSH-PX.

### 2.4. Rumen Function

The concentrations of VFAs and NH3-N were used as indicators of the rumen fermentation function and the effects of the dietary treatments on it [30]. Our data show that different levels of AFB1 contamination affected the concentrations of acetate, propionate, butyrate, valerate, isovalerate, and isobutyrate (all *p* < 0.05) (Table 3). However, there were no significant differences between the control and treatment groups in the acetate/propionate ratio. AFB1 significantly increased the concentration of rumen NH3-N (*p* < 0.05). The differences in NH3-N caused by AFB1 are shown in Figure 1d.

### 2.5. AFB1-Induced Metabolomic Changes

**Figure 2:**
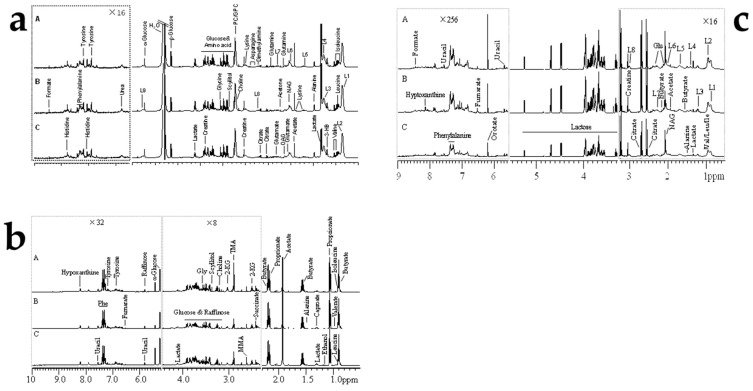
Representative 600 MHz 1D NOESY 1H-NMR spectra (δ 0.5–5.5 and δ 5.5–9.0) of rumen fluids (**a**), plasma (**b**), and milk (**c**) samples obtained from (**A**) control group, (**B**) AFB20 group, and (**C**) AFB40 group. The δ 5.5–9.0 region is magnified 16 times relative to the corresponding δ 0.5–5.5 region for the purpose of clarity. Key: Glu: glutamate; NAG: N-acetyl glycoprotein signals; L1: LDL, CH3-(CH2)n-; L2: VLDL, CH3-(CH2)n-; L3: LDL, CH3-(CH2)n-; L4: VLDL, CH3-(CH2)n-; L5: VLDL, -CH2; L6: lipid, -CH2-CH=CH-; L7: lipid, -CH2-C=O; L8: lipid, =CH-CH2-CH=.

**Figure 3:**
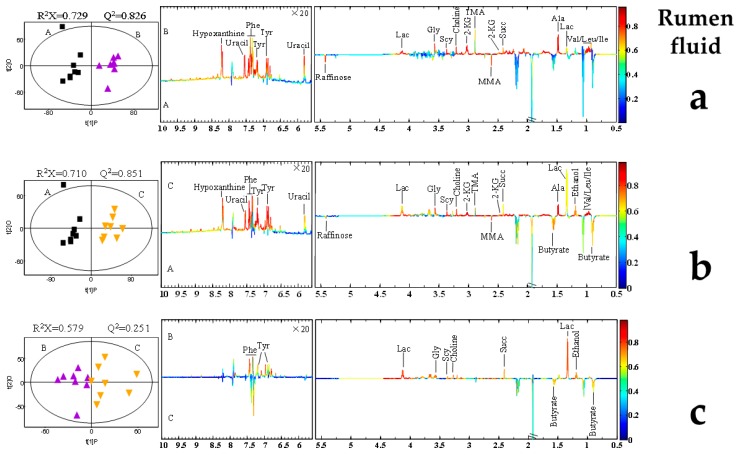
Orthogonal partial least-squares discriminant analysis (OPLS-DA) scores plot based on 1H NMR spectra of rumen fluid obtained from different groups. PLS-DA profiles between A and B (**a**); PLS-DA profiles between A and C (**b**); PLS-DA profiles between B and C (**c**). Each point on the score chart represents a sample. Control (A): black squares; AFB20 group (B): purple triangles; AFB40 group (C): yellow triangles.

**Figure 4:**
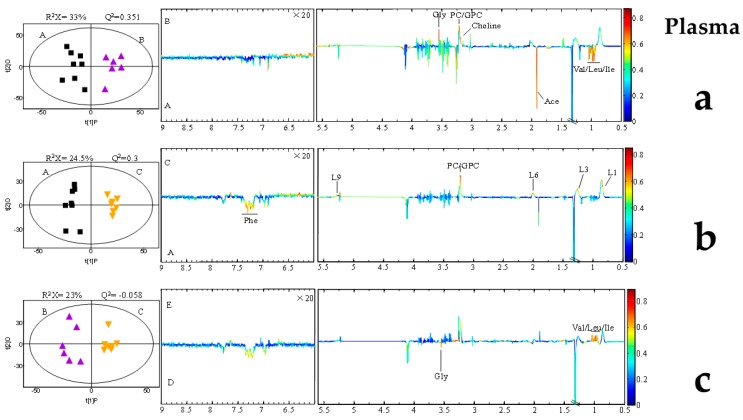
Orthogonal partial least-squares discriminant analysis (OPLS-DA) scores plot based on 1H NMR spectra of plasma obtained from different groups. (**a**) Partial least-squares discriminant analysis (PLS-DA) profiles between A and B; (**b**) PLS-DA profiles between A and C; (**c**) PLS-DA profiles between B and C. Each point on the score chart represents a sample. Control (A): black squares; AFB20 group (B): purple triangles; AFB40 group (C): yellow triangles.

**Figure 5:**
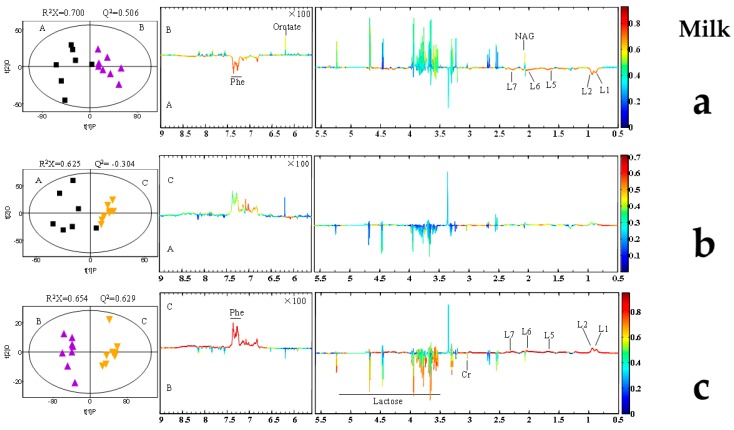
Orthogonal partial least-squares discriminant analysis (OPLS-DA) scores plot based on 1H NMR spectra of milk obtained from different groups. PLS-DA profiles between A and B (**a**); PLS-DA profiles between A and C (**b**); PLS-DA profiles between B and C (**c**). Each point on the score chart represents a sample. Control (A): black squares; AFB20 group (B): purple triangles; AFB40 group (C): yellow triangles.

Representative 600 MHz 1D NOESY 1H-NMR spectra (δ 0.5–5.5 and δ 5.5–9.0) for the rumen fluid, plasma, and, milk samples obtained from control group, AFB20 and AFB40 group were showed in Figure 2. The normalized NMR data for the rumen fluid, plasma, and, milk samples from the AFB1-treated and control animals at matched time points were analyzed with PCA (Figures S1–S3), PLS-DA (Figures S4–S6) and OPLS-DA (Figure 3, Figure 4 and Figure 5) for the individual biological matrices. The values for R2 and Q2 were used as the initial indicators of model quality, indicating the goodness of fit and the predictability of the models, respectively [6]. The AFB1 treatments caused significant changes in the 1H NMR profiles of the rumen fluid, plasma, milk relative to the control profiles, and recommendations based on cross-validated model parameters and replacement test results. The significantly altered metabolites were detected with OPLS-DA coefficient plots. Compared with the control, the AFB1 treatments significantly affected 19 metabolites in the rumen fluid, including butyrate, ethanol, succinate, phenylalanine, lactate, and tyrosine. In the plasma, the AFB1 treatments significantly affected 11 metabolites, including four lipids, acetate, phenylalanine, and choline. In milk, AFB1 exposure significantly influenced the levels of 9 metabolites, including five lipids, phenylalanine, creatine, etc. (Tables S2–S4).

## 3. Discussion

With advances in technological and analytical methods, metabolomics has been widely used to investigate the metabolites in body fluids or tissues extracts, and to understand the basic physiological and biochemical processes that respond to internal and external changes [6,16,17]. Mass-spectrometry-based metabolomics sensitively detects low-abundance metabolites. However, NMR-based metabolomics has been more widely used because the preparation of the samples is easy, and it is inexpensive, with good reproducibility [6,16]. These advantages are extremely convenient in the animal production industry for evaluating animal physiology and the metabolism of small molecular substances, and allow the systematic study of the toxicology of aflatoxins.

### 3.1. Effect of AFB1 on Milking Performance

Diets contaminated with mycotoxins can exert various effects, such as the depression of appetite, reduced feed intake, and reduced milk yield [2,8,31,32]. In the present study, there were no significant differences in feed intake or milk yield among the three groups (Figure 1a,b). These may indicate that AFB1 does not influence these phenotypic indices at the doses used. Ruminants are considered to be not very sensitive to mycotoxins than single-stomach animals, which is probably attributable to the detoxification of mycotoxins by rumen microorganisms [2,8,14]. Monogastric animals, such as swine and poultry, are susceptible to mycotoxins [33]. However, in another study, the consumption of AFB1 did not affect the dry matter intake or milk yield, however, it tended to reduce the FCM, milk fat production and milk protein concentration [34]. A diverse diet (including several types of feedstuffs, usually) and a large body mass may help dairy cows to resist mycotoxins [33,35]. The small amounts of mycotoxins consumed in the present study compared with the high overall feed intake may have ensured that the dilution effect rendered them relatively harmless [31]. There is evidence that increasing the aflatoxin content in cattle feeds (increase the concentration step by step from 0, 26, 56.4, 81.1, and 108.5 μg/kg) can reduces feed intake in a dose-dependent manner [36]. Therefore, the amount of aflatoxin consumed may have been a key factor affecting the feed intake in the present experiment. The lack of significant differences in the daily feed intake, milk yield, and milk composition in this study may indicate that the threshold for toxic effects was not reached (*p* > 0.05). Other mycotoxins studies have reported similar results [8,31,34]. For example, AFB1 contamination (20 μg/kg or 40 μg/kg) had no effect on dry matter intake, milk yield, or milk composition in dairy cows [8]. Other type of mycotoxin study has also been found natural contamination with *Fusarium* mycotoxins did not affect the dry matter intake, body weight, milk production, milk composition, or SCC in dairy cows during exposure to the mycotoxins or the addition of adsorbent [31].

Researchers have used physical, chemical, and biological methods to reduce mycotoxins in feedstuffs [8,31,37]. Detoxifying additives, such as modified yeast cell-wall extract, bentonite, and esterified glucomannan, has been shown to alleviate the toxic effects of mycotoxins in different livestock species [8,31,38] by binding to the mycotoxins so that they cannot be absorbed in the gastrointestinal tract. However, there has been no research into the effects of the interactions between detoxifying additives and mycotoxins [8,31].

### 3.2. Aflatoxin M1 Concentrations in Milk

Aflatoxins are the mycotoxins most intensively studied in dairy livestock [11]. As well as its lipophilic property, AFB1 is small, with a low molecular weight [39]. Therefore, it is rapidly absorbed by the gastrointestinal mucosa. Milk AFM1 is the monohydroxylated derivative of AFB1, and previous studies have suggested that the transformation of the AFB1 in feed to AFM1 in milk is mediated by cytochrome P450 [38,40]. AFM1 quickly appears in milk, at the first milking (1 h) after cows are fed AFB1-contaminated feed [38,39].

In the present study, the AFM1 concentration was measured during the addition and clearance periods (Figure 1c). but it did not exceed 400 ng/kg in milk, and the highest concentration during the experimental period was 393.35 ng/kg on day 7. After the ceased the AFB1, the level of AFM1 in the milk decreased to almost the same level as in the control group on day 7 of the clearance period. Previous studies have shown that 50% of the AFM1 excreted in milk can be detected within 12 h after pure AFB1 intake in goat [9,41]. Another study reported that AFM1 was continuously excreted in milk for several days (84 h) after a single dose of pure AFB1, with a carry-over rate of 0.032 μg/kg in dairy ewes [9]. The Food and Drug Administration (FDA) stipulates that the action level for AFM1 in milk is 0.5 μg/kg [42]. The maximum allowable concentration set by the European Commission (EC) is 0.05 μg/kg in milk [43]. In the present study, the consumption of AFB1 increased the concentration of AFM1 in milk, and the amount of AFM1 in some points exceeded the EC limit [34,43]. However, the amount of AFM1 in the milk did not exceed 0.5 μg/kg, so it was lower than both the China and FDA limits.

### 3.3. Effects of AFB1 on Serum Biochemical Parameters

Circular biochemical parameters can be used as indicators of basic physiological functions of body. For example, GGT, AST, ALT, and ALP are indicators of liver function [2,7,8]. In the present study, there were no significant differences in most serum biochemical parameters among the two AFB1 treatment groups and the control group. Similar results have observed in dairy ewes, dairy goats, and dairy cows [7,8,9].

The failure to detect differences in these biochemical indicators maybe attributable to the low amounts of AFB1 administered (20 or 40 μg/kg). It has been reported that when concentrations of 32 or 64 μg/kg AFB1 were added to dairy goat diets, there were no significant effects on serum biochemical parameters [7]. Other studies have confirmed that relatively low amounts of AFB1 had no effects on most blood biochemical indices [7,8,9]. A daily intake of 32 μg/day of pure AFB1 for 1 week did not alter the activities of several enzymes related to liver function in dairy sheep [10]. However, after a high dose of AFB1 (128 μg/day) was consumed for 2 weeks, the ALT activity of dairy sheep increased significantly [10]. These studies suggest that AFB1 has a dose-dependent effect on these biochemical parameters.

Immunoglobulins play a key defensive role in the body, and IgM, IgA, and IgG are important immune factors. They recognize and defend the organism against specific pathogens or foreign substances through the B-cell lymphatic system [44]. Previous studies have shown that AFB1 affects the immune systems of animals [2,34]. In the present study, IgM, IgA, and IgG did not differ significantly between the control and treatments. These data suggest that the immune functions remain stable during AFB1 exposure. Similar results have been reported in other studies [8,31]. However, several studies have demonstrated that the immune system is disturbed by mycotoxins. Dairy cows fed AFB1 displayed increased innate immune responses, with reduced concentrations of plasma haptoglobin (an indicator of innate immune stress) [34]. For example, one study found that haptoglobin increased in blood by 11.3 times after 12 h of lipopolysaccharides stimulation [45]. However, in other studies, researchers also found that bovine herpesviruses infections [46] or natural burdock virus [47] did not cause elevated blood concentration of haptoglobin. The administration of multiple mycotoxins also depressed serum IgA levels, whereas a single mycotoxin did not inhibit its function [7]. There are two possible reasons that no changes in immune parameters were detected in the present study. One possibility is that the parameter response to mycotoxins is reflected in other immune factors, such as haptoglobin [34]. Unfortunately, this index was not tested in the present study. Therefore, more measurements of immune function must be made to ensure more comprehensive results. The second possibility is the dose effect. Whereas both AFB1 (50 μg/kg) plus ochratoxin A (OTA; 100 μg/kg) and AFB1 (50 μg/kg) plus zearalenone (ZEA) (500 μg/kg) significantly suppressed serum IgA [7], when lower amounts of AFB1 (20 and 40 μg/kg) were fed to dairy cows, they had no significant effect on IgM, IgA, or IgG [8]. The same concentrations of AFB1 were also used in the present study.

Mycotoxins are important inducers of oxidative stress. A small amount of mycotoxin can spur the producing free radicals, and destroys its antioxidant capacity [48,49]. SOD, MDA, GSH-PX, and T-AOC are commonly used antioxidant indicators. In this study, the serum activity of SOD and the SOD/MDA ratio were reduced by treatment with AFB1, and the MDA and T-AOC concentrations were increased. SOD plays an important role in the conversion of oxygen radicals to peroxides [50]; MDA is a lipid peroxidation product [51]; and T-AOC reflects the total antioxidant levels of various antioxidants and antioxidant enzymes in an animal [7]. Therefore, significant changes in these parameters suggest that the dairy cows were in a state of oxidative stress. It was noteworthy that another study showed that treatment with OTA reduced the antioxidant capacity of rats [49].

### 3.4. AFB1-Induced Rumen VFAs and NH3-N Changes

Ruminal concentration of VFA and NH3-N were used as indicators of ruminal fermentation and effects of dietary treatment [30]. In the present study, data showed that different levels of contaminated AFB1 addition did affect he concentrations of acetate, propionate, butyrate, valerate, isovalerate, and isobutyrate (all *p* < 0.05) (Table 3). Moreover, AFB1 significantly increased the concentration of rumen NH3-N (*p* < 0.05) (Figure 1d). The possible reasons for this scenario are changed isoacids can promote utilization of microbial nitrogen to produce NH3-N leading to increased microbial protein synthesis. However, there were no significant differences between the control and treatment groups in the acetate/propionate ratio. This indicates that AFB1 does not alter the type of fermentation. However, some researchers believed that there are needs more verification of the function of the rumen [30].

### 3.5. AFB1-Induced Metabolomic Changes

In the present study, an AFB1 concentration of 20 μg/kg TMR was used as the low dose of AFB1. This approximates a safe dose (the content is lower than the 30 μg/kg, required by the China national standard GB13078-2017). The high AFB1 concentration of 40 μg/kg TMR was higher than the prescribed limit, and can be regarded as an unsafe dose.

In this study, we examined the metabolic changes that occurred in the rumen fluid, plasma, and milk after AFB1 exposure. Our results indicate that AFB1 exposure significantly affected the antioxidative functions and rumen functions. We also found residues of AFM1 in the cows’ milk. Therefore, we examined the comprehensive metabolic changes in the metabolic substances in the different matrices. The main purpose of this analysis was to investigate the systemic consequences of high and low levels of AFB1 exposure in dairy cows using an NMR-based metabolomic strategy. Similar studies have shown that T-2 toxin disrupts the normal metabolic processing of energy and nutrient substrates, and changes the lipids and proteins in different organs and organelles. These metabolic processes involve pathways of molecular metabolism and modification, such as deacetylation, hydroxylation, and oxidation [20,52]. Therefore, mycotoxins are likely to affect various energy- and nutrient-directed metabolic processes in the body [2,7,53].

#### 3.5.1. AFB1 Affects Amino Acid Metabolism

In this study, increasing levels of a range of amino acids were observed in the rumen fluid, plasma, and milk after AFB1 exposure (Figure 3, Figure 4 and Figure 5). Similar studies have been reported in other animals, including rats, piglets, dairy goats, and broiler chickens [6,16,25,54]. In the present study, AFB1 increased the levels of leucine, isoleucine, valine, alanine, glycine, phenylalanine, and α-ketoglutarate in rumen fluid. However, it reduced the levels of leucine, isoleucine, valine, and phenylalanine in plasma, and the level of phenylalanine in milk (Tables S2–S4). These changes imply that the amino acid metabolism in biofluids is significantly disturbed by AFB1 in dairy cows.

The concept of “functional amino acids” refers to those amino acids that participate in or mediate important metabolic pathways, thus influencing the health, survival, and reproduction of an organism [37]. Leucine independently regulates protein synthesis and catabolism in cells by activating the mammalian target of rapamycin (mTOR) signaling pathway [55,56]. Phenylalanine, which plays a very important physiological regulatory function in vivo, was the only metabolite significantly altered by AFB1 in all three biofluids in our present work. Normally, the main metabolic processes of phenylalanine include its merge into polypeptide chains, and its hydroxylation to tyrosine by the tetrahydrobiopterin-requiring phenylalanine hydroxylase reaction [37,57,58]. Phenylalanine is also an aromatic amino acid that promotes the secretion of cholecystokin (CCK) via calcium-sensing receptors [59]. CCK then induces the synthesis and release of enzymes in the gastrointestinal tract [60]. In ruminants, phenylalanine can be synthesized by anaerobic bacteria from the phenylacetic acid in the rumen, and increases the plasma insulin and CCK concentrations [57,60,61]. Phenylalanine is also a precursor of several amino acid metabolites. For example, tyrosine can be synthesized from phenylalanine by rumen bacteria [58]. Tyrosine plays an important role in protein biosynthesis, and as an intermediate in the biosynthesis of the specialized neurotransmitters produced from amino acids, such as dopamine, norepinephrine, and epinephrine [37,58]. In phenylalanine catabolism, cell metabolism first moves towards the pathway of tyrosine biosynthesis. Previous studies in animals and humans have suggested that phenylalanine influences protein metabolism. However, there has been less research in ruminants. Several studies have shown that the perfusion of phenylalanine into the intestines improves the secretion of pancreatic α-amylase, the main enzyme that decomposes starch [58,60]. Based on the parameters examined in the present study, several amino acids are affected by AFB1, which may indicate that protein metabolism is affected. Therefore, the measurement of the enzymes involved in protein synthesis and decomposition should be useful in understanding the effects of aflatoxins on the metabolism of amino acids. 1-methyl-histidine (1-MH) is a good indicator for monitoring protein metabolism. Researchers have developed a HPLC-MS/MS procedure that can accurately detect 1-MH in blood of dairy cows, and to estimate the animal’s protein metabolism [62]. Researchers suggested that it is better to use targeted metabolomics for research in amino acid metabolomics, based on the LC-MS/MS assay [63].

In general, the detection accuracy of NMR is not as good as that of mass spectrometry. However, there are still researchers here using the NMR-based method to detect changes of 1-MH in rat’s urine [64]. Unfortunately, no urine metabolomics testing was performed in this study. Although the present work checked blood metabolomics based on the NMR method, we did not find the change of 1-MH. But, this direction may be an important direction for future research.

AFB1 may causes various health problems in ruminants, and the effects of mycotoxins on mitochondrial functions and cell apoptosis are receiving increasing attention. AFB1 significantly impairs mitochondrial functions, and increases the generation of free radicals, induces cell apoptosis, and affects the NRF2 signaling pathway through the mitochondrial ROS-dependent signaling pathways [53]. A previous study reported that the ATP synthesis pathway was affected in chicks fed mycotoxin (2 mg AFB1/kg body weight), reducing both energy production and gene expression [52,54]. Several amino acids can be used as markers of energy metabolism. For example, α-ketoglutarate is an important metabolic intermediate in the microbial tricarboxylic acid cycle. It is a product of glutamic acid deamination, and the key intermediate connecting carbon and nitrogen metabolism. Two pathways downstream from α-ketoglutarate involve carbohydrate metabolism: one produces carbohydrate, mediated by malate and phosphoenolpyruvate carboxykinase, and the other produces carbonate, which is involved in gluconeogenesis [65]. A previous study demonstrated that changes in lactate and citrate can be used to determine whether the cori and krebs cycles are normal during energy metabolism, respectively [16]. Consistent with findings in broiler chickens [66] and ducks [67]. Study suggested that dairy goats fed a diet supplemented with 50 μg/kg AFB1 displayed fold changes in their serum glucose levels, indicating that the goats’ liver function had been damaged [16]. This result of AFB1 exposure may also involve in glycogenolysis. The depletion of hepatic glucose has also been observed in rats fed AFB1(0.32 mg/kg body weight per day) [6]. Therefore, the disturbance of glucose metabolism may be another consequence of mycotoxin exposure. However, in the present study, there was no direct evidence that carbohydrate metabolism was affected by AFB1, although there were significant changes in the glycemic amino acids (alanine, valine, isoleucine, glycine, and α-ketoglutarate), which are important components of the tricarboxylic acid cycle and the glucogenic process.

#### 3.5.2. AFB1 Affects Lipid and Nucleic Acid Metabolism

Significant changes were observed in the VFAs, lactate, and choline in the rumen fluid; in acetate, choline, and four other lipids in the plasma; and in creatine, orotate, and other lipids in the milk (Tables S2–S4). Several studies have reported altered levels of ketone bodies, such as acetoacetate, acetone, and 3-β-hydroxybutyrate, in AFB1-exposed dairy livestock [7,8,52]. These metabolites are the products of the β-oxidation of fatty acid in the mitochondria, and their changes suggests that the fatty acid β-oxidation is influenced by AFB1. However, we did not investigate the possible changes in ketones. Interestingly, our results showed no changes in feed intake or milk yields. This may indicate that mycotoxins do not affect energy metabolism from the macro-perspective of the energy balance. Significant changes in milk creatine have been detected in dairy cows fed AFB1, and the accumulation of blood creatine may be attributable to the accelerated conversion of phosphocreatine to creatine after AFB1 exposure [16]. In another study, the reduced levels of citrulline imply that the urea cycle is disturbed by AFB1, as citrulline is an intermediator. However, this is different from the AFB1-induced downregulated expression of the carbamoyl-phosphate synthetase I gene in mice [6]. Some studies found that mycotoxins alter metabolic enzymes, including the upregulation of peroxisome proliferator-activated receptor R, a key regulator of lipid metabolism [6,68]. Molecular biomarkers should allow us to clarify the effects of mycotoxins on lipid metabolism in future studies.

Branched-chain fatty acids are involved in the utilization of fiber by rumen microorganisms [30,57]. The tricarboxylic acid cycle may be disrupted by AFB1 exposure, which may affect energy metabolism [16]. When glucose is insufficient, the lipid stored in the body is used as the main metabolic substrate. However, lipid oxidation generates hydrogen peroxide, which may explain the free-radical-induced oxidative damage caused by AFB1 and other mycotoxins. Studies suggest that the oxidative stress induced by the lipid oxidation that occurs during AFB1 exposure triggers an antioxidative response [69]. Here, we observed elevated levels of serum MDA and T-AOC, and reductions in the serum activity of SOD and the SOD/MDA ratio after animals were fed diets contaminated with AFB1. These phenomena indicate both the effects of oxidative stress on the animals and the possibility that lipid metabolism are involved. Elevated levels of glutathione have been observed in the livers of rats exposed to AFB1, confirming that AFB1 activates antioxidative responses in the body. This is inconsistent with our data based on this index. A previous study suggested that AFB1 induces the upregulation of GGT and glutathione s-transferase in rats [70], and other genes may be involved in this process. For example, AFB1-induced oxidative stress may be associated with the upregulation of diaphorase and hemeoxygenase in rats and chicks [70,71]. Therefore, other indicators of gene expression under oxidative stress must be examined to explain these discrepancies.

Nucleic acids are involved in many biochemical processes in organisms. For instance, nucleic acids are precursors in the synthesis of biological macro-molecules such as ribonucleic acid (RNA) and deoxyribonucleic acid (DNA). ATP is the crux of energy metabolism in cells and play a key role in cellular energy metabolism. In this research, the increased lactate concentration in the rumen fluid after AFB1 exposure suggested shifts in both the cori and krebs cycles in response to changes in energy substrates [61]. A previous report indicated that biochemical parameters in the blood and urine can be used as reliable biomarkers of oxidative stress when different animals are fed diets contaminated with mycotoxins [2,20,72]. We also found significant differences in several serum oxidative biomarkers, such as SOD, T-AOC, and etc. However, few studies have examined reliable antioxidative indicators in rumen fluid. Interestingly, in the present study, the levels of hypoxanthine and uracil in the rumen differed significantly between the exposed and control cows. This purine nucleotide is decomposed in cells to produce hypoxanthine and xanthine, and ultimately produces uric acid under xanthine oxygenate catalysis. Nucleotide acids are commonly used as useful markers to quantify the levels of rumen microbial proteins [73,74]. Another study using a metabolomics approach suggested that grain diets increase the concentrations of hypoxanthine and uracil in the rumen fluid of dairy cows, especially diets with high proportions of barley [75]. Therefore, the increasing of hypoxanthine and uracil in the rumen fluid of cows on high-grain diets seems to be resulting from the changes in the rumen microflora [75,76]. Furthermore, when bacterial nucleic acid (RNA or DNA) is incubated with rumen fluid, it is rapidly converted to hypoxanthine, xanthine and uracil. [23]. Based on these findings, the rumen microbiota may be affected by mycotoxins, which would alter the nucleotide metabolism in the rumen. Thus, hypoxanthine and uracil appear to be biomarkers of adaptive changes in the rumen during AFB1 exposure in the present study.

### 3.6. Putative Analysis of Metabolites from Rumen Fluid, Plasma, and Milk

**Figure 6:**
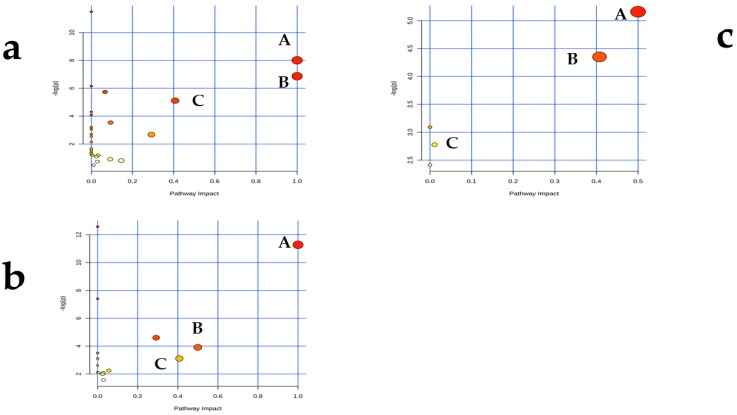
Metabolomic maps of the common metabolites identified in rumen fluid, plasma, and milk from dairy cows fed diets contaminated with AFB1. The x-axes represent the pathway impact and the y-axes represent the pathway enrichment. Larger sizes and darker colors represent greater pathway enrichment and greater pathway impact values, respectively. (**a**) Rumen fluid **A**. Valine, leucine, and isoleucine biosynthesis; **B**. Phenylalanine, tyrosine, and tryptophan biosynthesis; **C**. Phenylalanine metabolism. (**b**) Metabolomic pathway map of plasma. **A**. Valine, leucine, and isoleucine biosynthesis; **B**. Phenylalanine, tyrosine, and tryptophan biosynthesis; **C**. Phenylalanine metabolism. (**c**) Milk. **A**. Phenylalanine, tyrosine, and tryptophan biosynthesis; **B**. Phenylalanine metabolism; **C**. Arginine and proline metabolism.

The intake of AFB1 affects many physiological processes in animals. However, there were no significant differences in the acetate/propionate ratios of the control and treated cows, indicating that AFB1 did not change the fermentation type in the rumen [8,30,75]. Therefore, the physiological adjustments must happen elsewhere, rather than the type of rumen fermentation. These physiological adjustments may involve the metabolic pathways of several amino acids [2,75]. In the present study, the metabolic pathways were analyzed in different biofluids based on the corresponding metabolites. The three most strongly affected metabolic pathways were valine, leucine, and isoleucine biosynthesis, phenylalanine, tyrosine, and tryptophan biosynthesis, and phenylalanine metabolism in the rumen fluid and plasma; and phenylalanine, tyrosine, and tryptophan biosynthesis, phenylalanine metabolism, and arginine and proline metabolism in the milk. Therefore, the metabolic pathways altered in the different biofluids were very similar, only from the metabolic pathways listed here (Figure 6). These suggests that changes in the amino acid pathways are the most important effects of AFB1 consumption.

**Figure 7:**
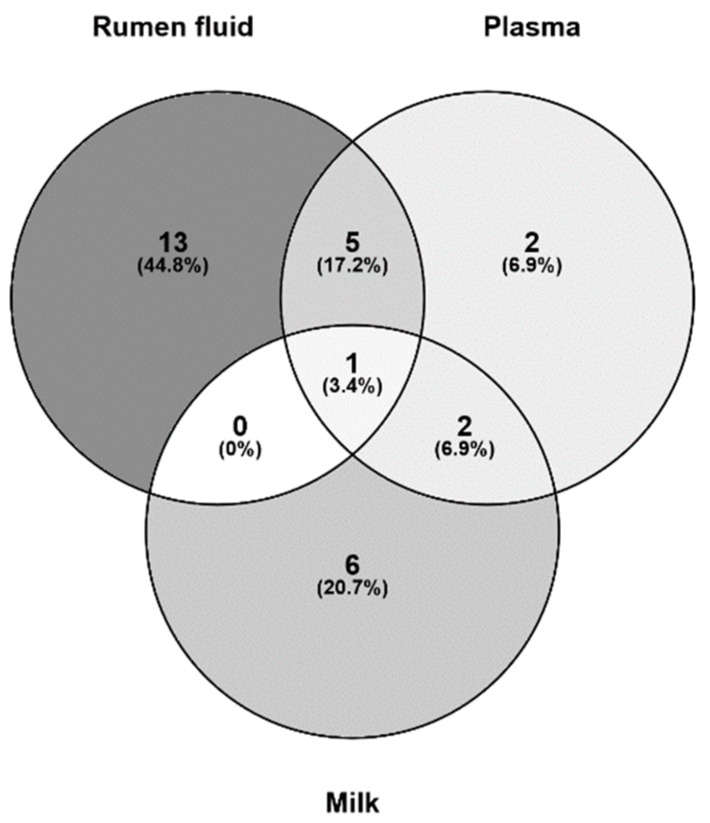
Unique and common metabolites identified in the rumen fluid, milk, and plasma from dairy cows fed diets contaminated with AFB1.

We found that phenylalanine was the only metabolite altered significantly in all three body fluids (Figure 7). A previous study also found that phenylalanine was significantly affected by AFB1 in rats [6]. In the theory of animal nutrition; phenylalanine is classified as an “essential” amino acid because it cannot be synthesized directly in animal cells. Therefore, dietary intake is the only source of essential amino acids. Once phenylalanine enters the body circulation, most is oxidized to another essential amino acid, tyrosine, by phenylalanine hydroxylase. The remaining phenylalanine then combines with tyrosine to synthesize neurotransmitters and hormones, and participates in glucose and fat metabolism in the body [77,78]. Several non-essential amino acids (α-ketoglutarate, pyruvate, oxaloacetic acid, and 3-glycerol phosphate) are the precursors in simple syntheses. Branched-chain amino acids (isoleucine, leucine, valine, etc.) are important in the synthesis of milk proteins [77]. Isoleucine decomposes into glucose, increasing the level of glucose and preventing protein damage. These observed changes suggest that AFB1 disrupts the regulation of the genes involved in amino acid metabolism [54,71]. Disorders of amino acid metabolism cause disease. Phenylalanine, tyrosine, and tryptophan are aromatic amino acids. A congenital lack of phenylalanine hydroxylase blocks the major metabolic pathway in which phenylalanine is hydroxylated to tyrosine. Because phenylalanine is required for many secondary metabolic pathways, including the generation of phenyl pyruvic acid from ammonia, this lack of hydroxylase activity increases the phenylpyruvic acid content in the blood and urine, which is phenylketonuria [79]. Studies have shown that modified phenylalanine can be used as a carrier of anticancer drugs, not only inhibiting the growth of cancer cell, but also reducing the side effects of the drugs [80]. Increased phenylalanine in the rumen fluid may reflect the physiological effects of AFB1 intake, and in turn, this increase in phenylalanine may reduce the toxicological effects of AFB1. A possible explanation of the reduced concentrations of phenylalanine in the blood and milk is that the detoxification processes in the rumen require large amounts of phenylalanine, which is co-opted from the phenylalanine reserves in the other two body fluids. The process of phenylalanine degradation may involve changes in antioxidative enzymes. Glutathione transferase plays a vital role in the cellular detoxification, as well as against oxidative stress. The zeta isoform of glutathione s-transferase is a bi-functional enzyme, also involved in the metabolic degradation of phenylalanine and tyrosine [81]. All these speculations are based on the results of existing studies, and the real reasons for the changes in phenylalanine in body fluids require further experimentation. For example, the phenylalanine concentration in rat liver extract was significantly affected by AFB1 [6], suggesting that studies of metabolic organs and cells will extend our understanding of the effects of AFB1 exposure.

## 4. Conclusions

Summarily, in this present study, we have not only detected the effects of AFB1 on animal health from a biochemical perspective, but also found some potential metabolic markers in rumen fluid, milk, and blood. Thus, our results suggest that it is important to study not only the macro-indicators (milk composition and production) but also micro-indicators (biofluids biomarkers) of mycotoxicity when assessing the risk that mycotoxins posed on dairy cows.

## 5. Materials and Methods

### 5.1. Animal Handling and Sample Preparation

The animal protocol (Protocol Number: IAS15020; Date of approval: 20150716) was approved by the Animal Care and Use Committee of the Institute of Animal Science, Chinese Academy of Agricultural Sciences. The experiments were conducted at Ningxia Helan Sinofarm Dairy Farm (Ningxia, China). The experiment included the addition of AFB1 to feed for a period of 7 days, followed by a clearance period of 7 days. In total, 24 multiparous Holstein cows in late lactation (lactation length: 283 ± 22 days; milk yield: 21.1 ± 2.6 kg/day; parity: 2.5–3.5) and with similar genetic backgrounds were used. The total mixed ration (TMR) (Table S1) was formulated to meet the nutritional requirements of the cows, as stipulated by the Feeding Standards of Dairy Cattle in China [82]. A concentration of 20 μg/kg or 40 μg/kg AFB1 in the basal TMR was used as the target concentration, selected with reference to the limitation prescribed by the national hygiene standard for animal feeds [29].

The cows were assigned to three groups (*n* = 8 individuals/group). The cows in the control group were fed an uncontaminated TMR with no AFB1. In the AFB20 group, the cows were fed the same diet as the control group, but 20 μg/kg AFB1was added to the TMR (dissolved in methanol). The cows in the AFB40 group were fed the same diet as the control group, but 40 μg/kg AFB1was added to the TMR. The daily feeding times were 00:30, 08:30, and 16:30. Milk was sampled three times a day (at 00:00, 08:00, and 16:00), and the individual milk yields were recorded on each occasion throughout the experimental period.

### 5.2. Experimental Methods and Sample Collection

The diets and residual feed were weighed and sampled every day during the period of AFB1 exposure and clearance. They were dried at 65 °C for 72 h, and then stored at −20 °C for later analysis. Analysis of the feed nutrients and mineral ions showed that it included crude protein, fat, calcium, phosphorus, ash, non-fiber carbohydrate, and neutral detergent fiber.

Milk was sampled three times per day (08:00, 16:00, and 00:00), and the yield was recorded individually per sampling time and per day throughout the experimental period. The milk samples from each cow per day were then mixed completely and 3 parallel samples were collected from it (about 50 mL each). Bronopol preservative (Broad Spectrum Microtabs II D&F Control System Inc., Dublin, CA, USA) was added to one aliquot, which was then sent to the testing center (Dairy Herd Improvement, Ningxia, China) to be analyzed for milk fat, milk protein, milk lactose, total solids, urea, and somatic cell count (SCC), with a CombiFoss™ FT+ instrument (Foss Electric, Hillerød, Denmark). The values for the 3.5% fat-corrected milk (FCM) yield were calculated according to the equation: FCM = (0.4324 × milk yield) + (16.218 × milk fat). The values for energy-corrected milk (ECM) were calculated according to the equation: ECM = ([milk yield × 0.383% milk fat] + 0.242% milk protein + 0.7832)/3.1138 [83]. The remaining two sets of samples were stored at −70 °C until analysis.

Blood samples were collected from the left jugular vein after milking on day 7, using sampling tubes with covers (with or without anticoagulant). The samples were allowed to settle for about 1.5 h before centrifuge at 3000× *g* for 20 min at 4 °C to isolate the serum, which was frozen at −70 °C for later analysis. The serum samples were analyzed for routine biochemical parameters with an Auto-Analyzer 7020 (Hitachi High-Technologies Corp., Tokyo, Japan) with colorimetric commercial kits (DiaSys Diagnostics Systems GmbH, Holzheim, Germany) for alanine aminotransferase (ALT), aspartate aminotransferase (AST), γ-glutamyl transpeptidase (GGT), alkaline phosphatase (ALP), total protein, albumin, globulin, albumin/globulin ratio (A/G), urea, creatinine, uric acid, total bilirubin, direct bilirubin, indirect bilirubin, triglyceride, and total cholesterol. Another set of serum sample was sent to Beijing CIC Clinical Laboratory (Beijing, China) for the determination of immune and antioxidant indices. The concentrations of immunoglobulin M (IgM), immunoglobulin A (IgA), and immunoglobulin G (IgG) were determined with bovine-immunoglobulin-based ELISA kits (Shanghai Meilian BioTech Company, Shanghai, China). The methods used to determine the levels of T-AOC, SOD activity, glutathione peroxidase (GSH-PX), and MDA have been reported in previous studies [7,8].

The rumen fluid was collected with an oral stomach tube about 1 h after the morning feed on day 7. The first 50–100 mL of liquid flowing from the tube was discarded to avoid contamination with saliva, and the oral stomach tube was washed twice with different water after each cow was sampled [84].

### 5.3. 1H NMR Spectroscopic Analysis of Milk

Deuterium oxide and deuterated chloroform were purchased from USA Cambridge Isotope Laboratories, Inc. (Tewksbury, MA, USA), and 3-(trimethylsilyl) propionic-2,2,3,3,D_4_-propionic acid sodium salt was purchased from Merck Canada Inc. (Kirkland, QC, Canada). High-performance liquid chromatography (HPLC)-grade methanol, methyl tert-butyl ether, water, formic acid, and ammonium formate were purchased from Merck (Darmstadt, Germany).

At the time of the NMR analysis, 24 milk samples collected on day 7 of the AFB1 treatment period were thawed at room temperature. To remove the milk fat, the milk samples were homogenized and centrifuged. The milk (500 μL) was mixed with 170 μL of deuterium oxide (D_2_O), and then centrifuged at 12,000× *g* for 10 min at 4 °C. Aliquots (500 μL) of the supernatants were transferred into 5 mm NMR tubes. All NMR spectra were obtained and recorded with a Bruker Avance III 600 spectrometer, operating at a 1H frequency of 600.13 MHz, and equipped with an ultra-low-temperature probe (Bruker BioSpin GmbH, Rheinstetten, Germany). The experimental parameters were as: spectral width, 12,000 Hz; waiting time, 2 s; mixing time, 100 ms; and sampling number, 32 K. The NMR spectra were manually phased, baseline-corrected, and referenced to trimethylsilylpropanoic acid (CH_3_, δ 0.00) using the Bruker Topspin 3.0 software (Bruker GmbH, Karlsruhe, Germany). The NMR spectra were visually inspected with Amix 3.9.13 (Bruker, Biospin, Italy). Finally, the NMR spectra were integrated over a range of 0.50–9.50 ppm, using an interval of 2.4 Hz, and the water peak (δ 5.05–4.75) was removed.

### 5.4. 1H NMR Spectroscopic Analysis of Plasma

Blood (2 mL) was collected on day 7 of the AFB1 treatment period and placed in heparinized eppendorf tubes. The plasma was separated by centrifugation and frozen before the NMR measurements were made. The plasma sample was thawed at room temperature, and 200 μL of the plasma sample was placed in 1.5 mL tube, and 400 μL of buffer (45 mM NaH_2_PO_4_/K_2_HPO_4_; 0.9% NaCl; pH: 7.4; 50% D_2_O) was added. After shaking and mixing, centrifuge (4 °C, 16,099× *g*, 10 min) Take 500 μL of supernatant to 5 mm nuclear magnetic tube, and inverted several times to ensure mixing well. Plasma samples were subjected to 1H NMR detection using Bruker AVIII 600 MHz NMR (proton resonance frequency 600.13 MHz, and ultra-low temperature probe). Detection of small molecule metabolites in each sample using a 1D Carr–Purcell–Meiboom–Gill (CPMG) spin-echo pulse sequence with pre-saturated pressurized water The FID signal of all the 1H NMR spectra collected was subjected to an exponential window function with a broadening factor of 1 Hz, followed by Fourier transform to improve the signal-to-noise ratio, and then manually performed spectral phase and baseline correction, and NMR of all samples. The spectrum uses a bimodal calibration at the low field of α-glucose (δ 5.23). The NMR spectra were integrated using the relevant software. The parameters were as follows: the integration interval was 9.0–0.5 ppm, the integration interval was 0.002 ppm, the water peak (5.20–4.20 ppm), and the urea peak (5.60–6.00 ppm) were removed. Normalized multi-variable data after normalizing the integrated data.

### 5.5. 1H NMR Spectroscopic Analysis of Rumen Fluid

The rumen samples were thawed at room temperature and centrifuged at 10,000× *g* for 140 min to remove macro-molecules (fine feed particles and microbiota). The supernatant was collected and passed through a 0.2 μM syringe filter (Fischer Scientific, Fairlawn, NJ, USA). Then 35 μL of D_2_ O and 15 μL of a standard buffer solution (0.16 mM disodium-2,2-dimethyl-2-silapentane-5-sulfonate, 10 mM imidazole, and 0.02% NaN_3_ in H_2_O) were added to 300 μL of filtered rumen sample. These samples (350 μL) were transferred to a standard Shigemi microcell NMR tube for NMR spectral analysis.

Aliquots (500 μL) were decanted into 5 mm NMR tubes and inverted several times to ensure thorough mixing. The 1 H NMR spectrum was acquired for each sample at 600.13 MHz on a Bruker Avance III 600 spectrometer, operating at a 1 H frequency of 600.13 MHz, and equipped with an ultra-low-temperature probe. 1 D NMR was performed with the standard solvent suppression pulse sequence, as described for the acquisition of MAS NMR spectra. For each sample, 64 transients were collected into 64 K data points, with a relaxation delay of 2 s and a mixing period of 100 ms. A spectral width of 9600 Hz and an acquisition time per scan of 3.41 s were used. The 1 D CPMG spin-echo pulse sequence, using a fixed total spin–spin relaxation delay of 80 ms, was used to measure the spin-echo 1 H NMR spectra of all samples. NMR spectra were integrated by software, and the used parameters were listed here: the integration window was 10–0.5 ppm, and the interval was 0.002 ppm, and the water peak (5.20–4.45 ppm) was removed. After the integration is completed, the data needs to be normalized, and then the multivariate data statistical analysis is performed.

### 5.6. Data Analysis 

Data on feed intake, milk yield, milk composition, serum biochemical parameters, serum antioxidant and immune indices, rumen fluid VFAs, and NH3-N were analyzed with repeated-measures ANOVA in SPSS Statistics and post-hoc tests (IBM SPSS Statistics v19.0, SPSS Inc., Chicago, IL, USA). The statistical model included the treatment as the fixed effect, and the cows within each treatment group as the random effect. The data on feed intake, milk yield, and milk composition before day 1 of the treatment period were used as a co-variate in the statistical analysis of each variable. Tukey’s adjustment was used to determine significant differences between the least squares means. All statements of statistical significance were based on a probability of *p* < 0.05.

The normalized data were analyzed with a multivariate analysis using the SIMCA-P+ software (v11.5, Umetrics AB, Umea, Sweden). The 1 H NMR spectra were first analyzed with a principal component analysis (PCA), based on mean center scaling to reflect overall differences. The spectra were then analyzed with a partial least-squares discriminant analysis (PLS-DA) and an orthogonal partial least-squares discriminant analysis (OPLS-DA), both of which are supervised methods. The quality of each model was determined with the goodness-of-fit parameter (R2) and the goodness-of-prediction parameter (Q2). The statistical significance of differences in metabolite concentrations and the appropriate correlation coefficients were determined with OPLS-DA.

## Figures and Tables

**Table 1:** Milk parameters of dairy cows fed diets contaminated with AFB1 ^1^.

Item	Control	AFB20	AFB40	SEM	*p* Value
Daily milk yield (kg/day)	17.23	16.94	17.9	5.33	0.29
Milk fat (%)	4.93	4.50	4.69	0.11	0.32
Milk protein (%)	4.01	4.13	4.17	0.08	0.48
Milk lactose (%)	5.08	5.06	5.05	0.06	0.98
Total solids (%)	15.17	14.73	14.97	1.17	0.58
Urea (mg/dl)	33.04	25.79	23.2	5.01	0.73
SCC (cells/mL) ^2^	0.27	0.21	0.22	0.05	0.28
FCM (kg) ^2^	16.19	17.03	16.54	0.58	0.59
ECM (kg) ^2^	7.36	7.67	7.42	0.41	0.85

^1^ Control group (AFB1 null); AFB20 group (20 μg/kg in the total mixed ration); and AFB40 group (40 μg/kg in the total mixed ration). ^2^ Feed efficiency (%), milk yield/DMI (dry matter intake); SCC, somatic cell count; FCM, 3.5% fat-corrected milk. Values for 3.5% FCM yield were calculated with the equation: (0.4324 × milk yield) + (16.218 × milk fat); ECM, energy-corrected milk. Values for ECM were calculated with the equation: (milk yield × 0.383% milk fat + 0.242% milk protein + 0.7832)/3.1138^30^.

**Table 2:** Effects of diets contaminated with AFB1 ^1^ on serum biochemical, antioxidant, and immune indices in dairy cows.

Item ^2^	Control	AFB20	AFB40	SEM	*p* Value ^3^
ALT (U/L)	29.75	26.00	26.38	1.02	0.26
AST (U/L)	72.75	79.14	75.88	3.65	0.80
GGT (U/L)	43.09	38.06	37.84	2.39	0.61
ALP (U/L)	90.31	96.56	68.33	15.83	0.76
TP (g/L)	37.53	41.64	39.30	1.15	0.54
ALB (g/L)	36.44	35.46	36.95	0.62	0.64
GLOB (g/L)	3.10	3.03	3.18	1.22	0.42
Urea (mmol/mL)	71.13	69.29	65.50	0.10	0.84
CR (μmol/L)	25.85	40.86	34.01	1.62	0.35
UA (μmol/L)	11.05	9.58	10.98	3.37	0.20
TBil (μmol/L)	2.28	2.03	2.39	0.57	0.53
DBil (μmol/L)	8.78	7.54	8.59	0.09	0.32
IBiL (μmol/L)	0.05	0.05	0.05	0.50	0.59
TG (mmol/mL)	6.03	6.60	6.57	0.00	0.83
TC (mmol/mL)	3.10	3.03	3.18	0.24	0.56
GSH-PX (U/mL)	760.5	714.0	683.25	37.8	0.71
MDA (nmol/mL)	6.61 ^a^	10.74 ^a^	13.17 ^b^	1.01	0.02
SOD (U/mL)	113.03 ^a^	109.02 ^a^	106.17 ^b^	1.04	0.01
SOD/MDA	18.49 ^a^	11.43 ^b^	9.91 ^b,c^	1.3	0.01
T-AOC (U/mL)	0.74 ^a^	2.96 ^b^	4.15 ^b,c^	0.39	<0.01
IgG (μg/mL)	13.12	11.91	12.7	1.03	0.90
IgA (μg/mL)	59.43	51.86	52.32	2.87	0.50
IgM (μg/mL)	22.4	23.64	22.24	1.24	0.89

^1^ Control group (AFB1 null); AFB20 group (20 μg/kg in the total mixed ration); and AFB40 group (40 μg/kg in the total mixed ration). ^2^ ALT, alanine aminotransferase; AST, aspartate aminotransferase; GGT, γ-glutamyl transpeptidase; ALP, alkaline phosphatase; TP, total protein; ALB, albumin; GLOB, globulin; A/G, albumin/globulin; CR, creatinine; UA, uric acid; TBil, total bilirubin; DBil, direct bilirubin; IBiL, indirect bilirubin; TG, total triglyceride; TC, total cholesterol; GSH-PX, glutathione peroxidase; MDA, malondialdehyde; T-AOC, total antioxidant capacity; SOD, superoxide dismutase; IgG, immunoglobulin G; IgA, immunoglobulin A; IgM, immunoglobulin M. ^3^ Probability associated with the F-test of treatment differences. ^abc^ Means in the same row with different superscript letters are significantly different (*p* < 0.05 or 0.01), as determined with Tukey’s test.

**Table 3:** Effects of diets contaminated with AFB1 ^1^ on concentrations of rumen volatile fatty acids in dairy cows.

Item (μg/mL)	Control	AFB20	AFB40	SEM	*p* Value
Acetate	65.55 ^a^	62.54 ^a,b^	53.62 ^c^	1.63	<0.01
Propionate	22.49 ^a^	22.75 ^a,b^	19.09 ^c^	0.59	0.01
Acetate/Propionate	2.93	2.77	2.81	0.05	0.44
Isobutyrate	12.73 ^a^	12.35 ^b^	9.73 ^c^	0.48	<0.01
Butyrate	1.27 ^a^	1.44 ^a,b^	1.08 ^c^	0.05	0.01
Isovalerate	1.39 ^a^	1.29 ^a^	0.99 ^a,b^	0.05	<0.01
Valerate	65.55 ^a^	62.53 ^a,b^	53.62 ^b,c^	1.62	<0.01

^1^ Control group (AFB1 null); AFB20 group (20 μg/kg in the total mixed ration); and AFB40 group (40 μg/kg in the total mixed ration). ^abc^ Means in the same row with different superscript letters are significantly different (*p* < 0.05 or 0.01), as determined with Tukey’s test.

## Supplementary Materials

The following are available online at http://www.mdpi.com/2072-6651/11/2/77/s1, Figure S1: PCA analysis based on 1 H NMR spectra of rumen fluid obtained from different groups, Figure S2: PCA analysis based on 1 H NMR spectra of plasma obtained from different groups, Figure S3: PCA analysis based on 1 H NMR spectra of milk obtained from different groups, Figure S4: PLS-DA scores plots based on 1 H NMR spectra of rumen fluid obtained from different groups, Figure S5: PLS-DA scores plots based on 1 H NMR spectra of plasma obtained from different groups, Figure S6: PLS-DA scores plots based on 1 H NMR spectra of milk obtained from different groups, Table S1: Ingredients of the total mixed ration, Table S2: Rumen fluid metabolites that differed significantly between groups and their correlation coefficients, Table S3: Plasma metabolites that differed significantly between groups and their correlation coefficients, Table S4: Milk metabolites that differed significantly between groups and their correlation coefficients.
