# Supplementary material for: Biological System Responses of Dairy Cows to Aflatoxin B1 Exposure Revealed with Metabolomic Changes in Multiple Biofluids
Source: Toxins (Basel). 2019 Feb 1;11(2):77. doi: 10.3390/toxins11020077 (PMC6410036; doi:10.3390/toxins11020077)
Supplement: Supplementary file 1 [file toxins-11-00077-s001.pdf]

Supplementary Materials: Biological System Responses of Dairy Cows to Aflatoxin B1 Exposure Revealed with Metabolomic Changes in Multiple Biofluids

Qian Wang, Yangdong Zhang, Nan Zheng, Liya Guo, Xiaoming Song, Shengguo Zhao and Jiaqi Wang

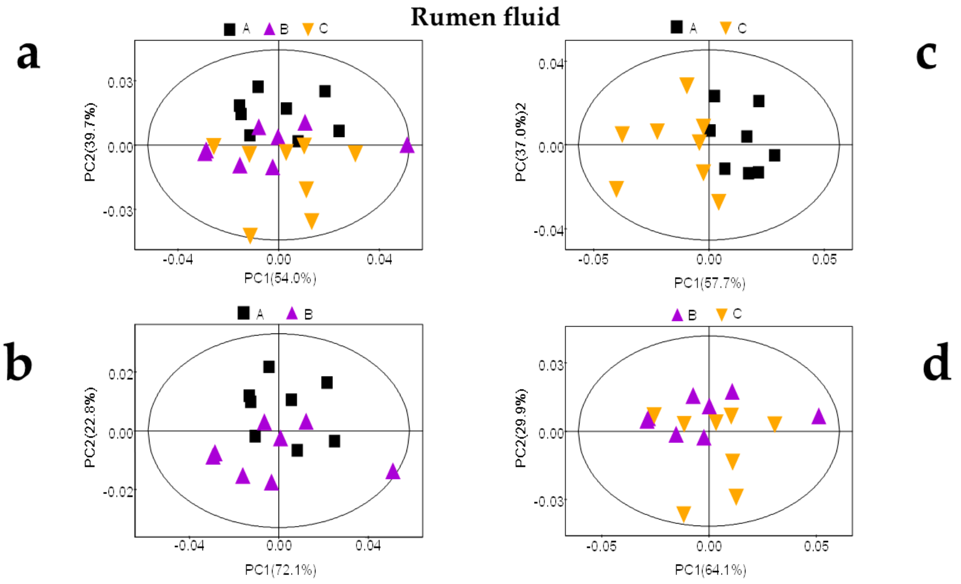

Figure S1. PCA analysis based on 1H NMR spectra of *rumen fluid* obtained from different groups. (**a**) PCA profiles among A, B and C; (**b**) PCA profiles between A and B; (**c**) PCA profiles between A and C; (**d**) PCA profiles between B and C. Each point on the score chart represents a sample. Control (A): black squares; AFB20 group (B): purple triangles; AFB40 group (C): yellow triangles.

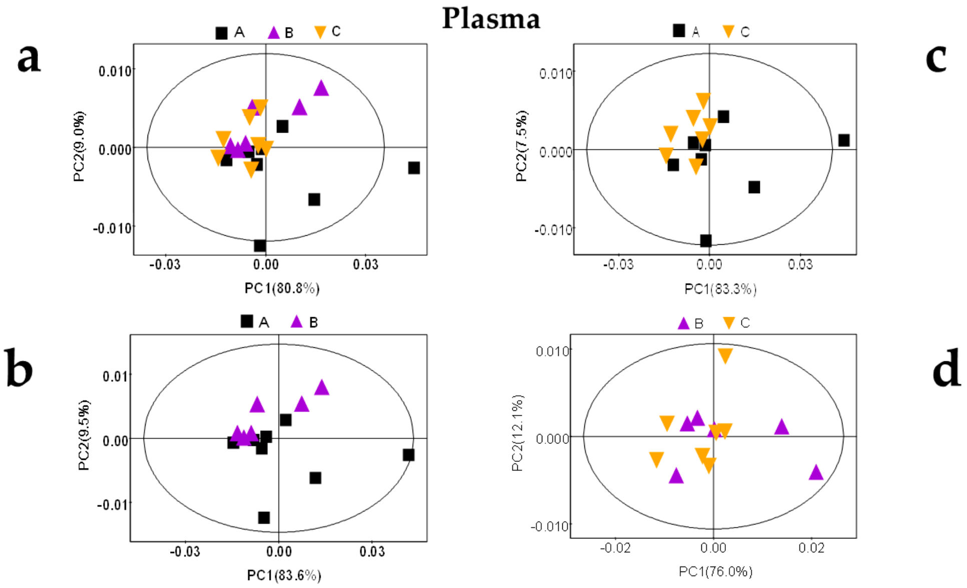

Figure S2. PCA analysis based on 1H NMR spectra of *plasma* obtained from different groups. (**a**) PCA profiles among A, B and C; (**b**) PCA profiles between A and B; (**c**) PCA profiles between A and C; (**d**) PCA profiles

between B and C. Each point on the score chart represents a sample. Control (A): black squares; AFB20 group (B): purple triangles; AFB40 group (C): yellow triangles.

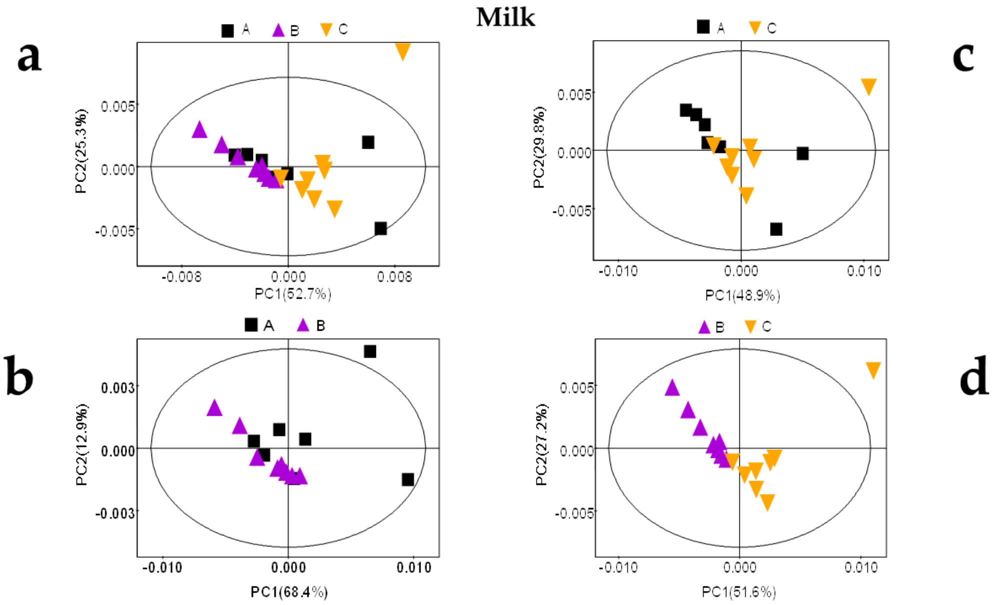

Figure S3. PCA analysis based on 1H NMR spectra of *milk* obtained from different groups. (**a**) PCA profiles among A, B and C; (**b**) PCA profiles between A and B; (**c**) PCA profiles between A and C; (**d**) PCA profiles between B and C. Each point of the score chart represents a sample. Control (A): black squares; AFB20 group (B): purple triangles; AFB40 group (C): yellow triangles.

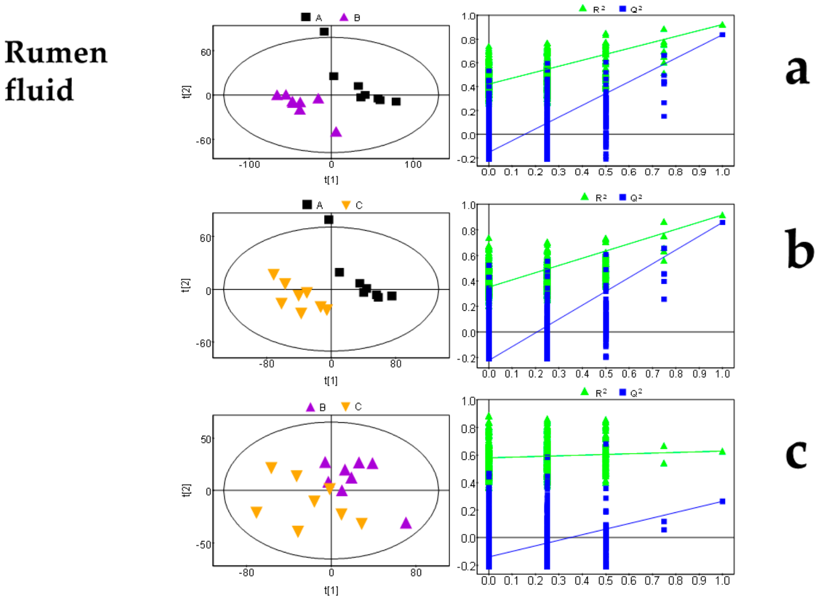

Figure S4. PLS-DA scores plots based on 1H NMR spectra of *rumen fluid* obtained from different groups. (**a**) PLS-DA profiles between A and B [R^2X = 72.9%, R^2Y = 0.923, Q^2 = 0.836; intercept: R^2 = (0.0, 0.43), Q^2 = (0.0, −0.015)]; (**b**) PLS-DA profiles between A and C [R^2X = 71%, R^2Y = 0.916, Q^2 = 0.858; intercept: R^2 = (0.0, 0.359), Q^2 = (0.0, −0.235)]; (**c**) PLS-DA profiles between B and C [R^2X = 57.9%, R^2Y = 0.627, Q^2 = 0.262; intercept: R^2 = (0.0, 0.591), Q^2 = (0.0, −0.134)]. Each point on the score chart represents a sample. Control (A): black squares; AFB20 group (B): purple triangles; AFB40 group (C): yellow triangles.

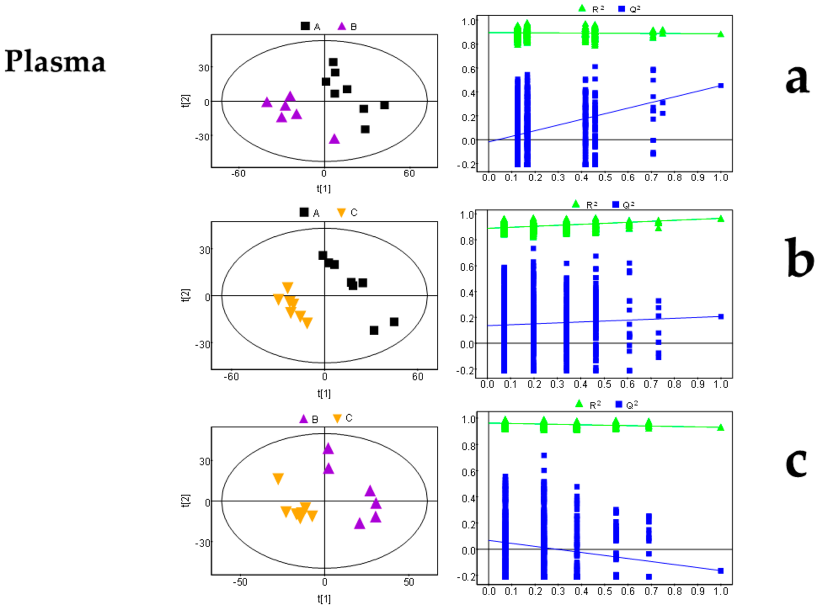

Figure S5. PLS-DA scores plots based on 1H NMR spectra of *plasma* obtained from different groups. (**a**) PLS-DA profiles between A and B [$R^2X = 33\%$, $R^2Y = 0.886$, $Q^2 = 0.452$; intercept: $R^2 = (0.0, 0.894)$, $Q^2 = (0.0, -0.0377)$]; (**b**) PLS-DA profiles between A and C [$R^2X = 24.5\%$, $R^2Y = 0.965$, $Q^2 = 0.205$; intercept: $R^2 = (0.0, 0.895)$, Q2 = (0.0, 0.17)]; (**c**) PLS-DA profiles between B and C [$R^2X = 23\%$, $R^2Y = 0.931$, $Q^2 = 0.164$; intercept: $R^2 = (0.0, 0.962)$, $Q^2 = (0.0, 0.0559)$]. Each point on the score chart represents a sample. Control (A): black squares; AFB20 group (B): purple triangles; AFB40 group (C): yellow triangles.

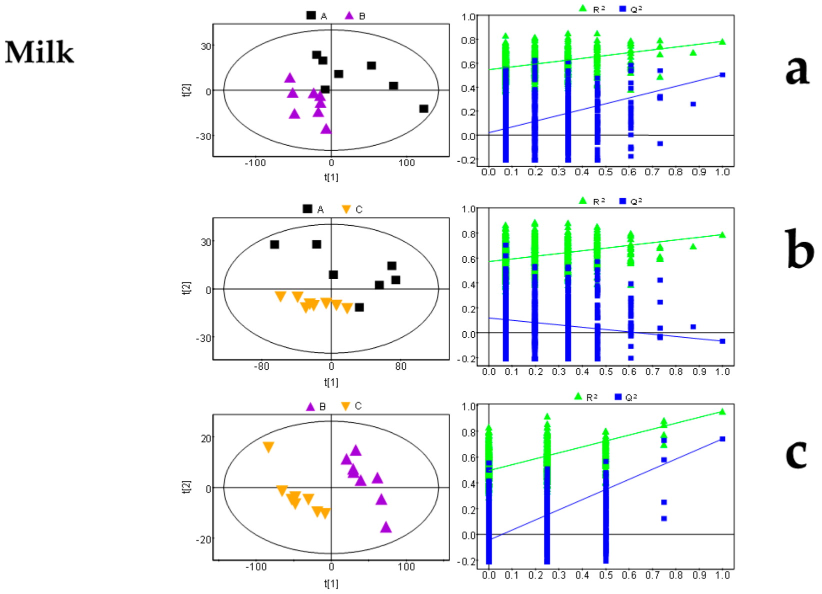

Figure S6. PLS-DA scores plots based on 1H NMR spectra of *milk* obtained from different groups. (**a**) PLS-DA profiles between A and B [$R^2X = 70\%$, $R^2Y = 0.781$, $Q^2 = 0.503$; intercept: $R^2 = (0.0, 0.553)$, $Q^2 = (0.0, 0.0165)$]; (**b**) PLS-DA profiles between A and C [$R^2X = 62.5\%$, $R^2Y = 0.787$, $Q^2 = (0.0, -0.0666)$; intercept: $R^2 = (0.0, 0.587)$, Q2 = (0.0, 0.16)]; (**c**) PLS-DA profiles between B and C [$R^2X = 65.4\%$, $R^2Y = 0.952$, $Q^2 = 0.\ 0.738$; intercept: $R^2 = (0.0, 0.506)$, $Q^2 = (0.0, -0.0519)$]. Each point on the score chart represents a sample. Control (A): black squares; AFB20 group (B): purple triangles; AFB40 group (C): yellow triangles.

Table S1. Ingredients of the total mixed ration [1].

Item	Amount (%)
Oat hay	2.24
Corn silage	45.57
Alfalfa hay	8.07
Soybean meal	4.86
DDGS[2]	5.58
Corn meal	11.88
Wheat bran	2.24
Extruded soy flour	0.56
Corn bran shotcrete	4.59
Cottonseed	4.48
Mineral meal	0.22
Diamond XP	0.13
$NaHCO_3$	0.54
NaCl	0.07
Premix	1.12
Water	7.84
**Nutrients, % of DM[3]**	
Crude protein	17.65
Fat	4.15
Nonfiber carbohydrate	39.5
Neutral detergent fiber	29.49
Calcium	0.8
Phosphorus	0.48
Ash	9.27
**Energy (mcal/Kg)**	
Metabolic energy	2.88
Net Energy	1.67

Notes: [1] Control group (AFB1 null); AFB20 group (20μg/kg in the total mixed ration); and AFB40 group (40μg/kg in the total mixed ration). [2] Dried distillers' grains with soluble. [3] Dry matter.

Table S2. Rumen fluid metabolites that differed significantly between groups and their correlation coefficients.

Metabolites [b]	r [a]		
	A vs. B	A vs. C	B vs. C
Val (valine): 0.98 (d), 1.02 (d)	0.870	0.897	-
Leu (leucine): 0.96 (d), 0.97 (d)	0.888	0.911	-
Ile (isoleucine): 0.94 (t); 1.00 (d)	0.808	0.957	-
Butyrate: 0.90 (t), 1.56 (m)	-	−0.681	−0.792
Ethanol: 1.18 (t)	-	0.748	0.743
Lac (lactate): 1.32 (d); 4.11 (q)	0.707	0.679	0.785
Ala (alanine): 1.48 (d)	0.862	0.893	-
TMA (trimethylamine): 2.88 (s)	0.673	0.733	-
Raffinose: 5.41 (d), 3.56 (m), 3.67 (d), 3.76 (m), 3.82 (m), 3.89 (m), 3.94 (t), 4.21 (d)	−0.843	−0.794	-
Succ (succinate): 2.41 (s)	0.800	0.669	0.704
MMA (methylamine): 2.61 (s)	−0.802	−0.842	-
Choline: 3.20 (s)	0.850	0.847	0.794
Scy (scyllitol): 3.36 (s)	0.902	0.817	0.702
Gly (glycine): 3.56 (s)	0.925	0.878	0.870
α-KG (α-ketoglutarate): 2.49 (t), 3.02 (t)	0.904	0.936	-
Uracil: 5.80 (d), 7.54 (d)	0.822	0.869	-
Tyr (Tyrosine): 6.91 (d), 7.18 (d)	0.864	0.920	0.717
Phe (phenylalanine): 7.32 (d), 7.37 (t), 7.42 (dd)	0.843	0.908	0.839
Hypoxanthine: 8.22 (s), 8.20 (s)	0.885	0.850	-

[a] Correlation coefficients: positive and negative signs indicate positive and negative correlations between concentrations, respectively. Correlation coefficient ∣r∣ > 0.666 was used as the cutoff value for statistical significance based on the discrimination significance at the level of $p = 0.05$ and *df* (degree of freedom) = 7. ″-″ means the correlation coefficient ∣r∣ < 0.666. [b] Multiplicity: s, singlet; d, doublet; t, triplet; q, quartet; dd, doublet of doublets; m, multiplet; br, broad resonance.

Table S3. Plasma metabolites that differed significantly between groups and their correlation coefficients.

Metabolites [b]	r [a]		
	A vs. B	A vs. C	B vs. C
Val (Valine): 0.98 (d), 1.04 (d)	−0.859	-	0.775
Leu (Leucine): 0.96 (d), 0.97 (d)	−0.848	-	0.881
Ile (Isoleucine): 0.94 (t); 1.00 (d)	−0.873	-	0.786
Ace (Acetate): 1.91 (s)	−0.778	-	-
Choline: 3.19 (s)	0.786	-	-
PC/GPC: 3.22 (s)	0.759	0.734	-
Gly (Glycine): 3.56 (s)	0.773	-	−0.776
Phe (Phenylalanine): 7.32 (d), 7.37 (t), 7.42 (dd)	-	−0.708	-
L1: Lipid, C<u>H</u>$_3$-(CH$_2$)$_n$-(LDL): 0.86 (br)	-	0.708	-
L3: Lipid, CH$_3$-(C<u>H</u>$_2$)$_n$- (LDL): 1.27 (br)	-	0.710	-
L6: Lipid, -C<u>H</u>$_2$-CH = CH-: 2.01 (br)	-	0.726	-
L9: Lipid, -CH = CH-: 5.27 (br)	-	0.732	-

[a] Correlation coefficients: positive and negative signs indicate positive and negative correlations in the concentrations, respectively. Correlation coefficient ∣r∣ > 0.666, 0.707 or 0.755 was used as the cutoff value for statistical significance based on the discrimination significance at the level of $p = 0.05$ and *df* (degree of freedom) = 7, 6, or 5, respectively. ″-″ means correlation coefficient ∣r∣ < 0.666, 0.707, or 0.755, respectively. [b] Multiplicity: s, singlet; d, doublet; t, triplet; q, quartet; dd, doublet of doublets; m, multiplet; br, broad resonance.

Table S4. Milk metabolites that differed significantly between groups and their correlation coefficients.

Metabolites [b]	r [a]		
	**A vs. B**	**A vs. C**	**A vs. B**
OAG (O-acetyl-glycoprotein): 2.05 (s)	-	0.779	-
Cr (Creatine): 3.03 (s), 3.93 (s)	-	-	0.691
Orotate: 6.20 (s)	-	0.669	-
Phe (Phenylalanine): 7.27 (d), 7.30 (t), 7.38 (dd)	-	−0.758	-
L1: Lipid, CH3-(CH2)n-(LDL): 0.86 (br)	-	−0.741	0.875
L2: Lipid, CH3-(CH2)n-(VLDL): 0.88 (br)	-	−0.766	0.895
L5: Lipid, -CH2-CH2-C = O (VLDL): 1.58 (br)	-	−0.689	0.902
L6: Lipid, -CH2-CH = CH-: 2.01 (br)	-	−0.719	0.891
L7: Lipid, -CH2-C = O: 2.23 (br)	-	−0.694	0.912

[a] Correlation coefficients: positive and negative signs indicate positive and negative correlations in the concentrations, respectively. Correlation coefficient $|r| > 0.602$ was used as the cutoff value for the statistical significance based on the discrimination significance at the level of $p = 0.05$ and *df* (degree of freedom) = 7. "-" means correlation coefficient $|r| < 0.666$. [b] Multiplicity: s, singlet; d, doublet; t, triplet; q, quartet; dd, doublet of doublets; m, multiplet; br, broad resonance.
